# Lupus IgG deposition causes arthritis but inhibits bone destruction through competitive occupation of FcγRI and reduced RANKL signalling

**DOI:** 10.1002/cti2.1174

**Published:** 2020-09-06

**Authors:** Wei Qiao, Huimin Ding, Yuyue Zuo, Lijuan Jiang, Jiayuan Zhou, Xiaoxiao Han, Likai Yu, Rong Du, Christian M Hedrich, Guo‐Min Deng

**Affiliations:** ^1^ Department of Clinical Laboratory The fourth affiliated hospital of Nanjing Medical University Nanjing China; ^2^ Department of Orthopedics BenQ Medical Center The affiliated BenQ Hospital of Nanjing Medical University Nanjing China; ^3^ Department of Rheumatology Union Hospital Tongji Medical College Huazhong University of Science and Technology Wuhan China; ^4^ Department of Women’s & Children’s Health, Institute of Translational Medicine University of Liverpool Liverpool UK

**Keywords:** arthritis, bone destruction, Fcγ receptor, IgG, osteoclastogenesis, systemic lupus erythematosus

## Abstract

**Objectives:**

Bone destruction is a remarkable feature of inflammatory arthritis. It remains unknown why arthritis associated with the systemic autoimmune/inflammatory condition systemic lupus erythematosus (SLE) does not result in erosion and destruction. We aimed to determine the role of autoantibody in the pathogenesis of non‐erosive arthritis in SLE.

**Methods:**

We analysed medical record of SLE patients, investigated whether autoantibody induces arthritis lacking bone destruction in animal models and determined whether SLE autoantibody inhibits osteoclastogenesis induced by RANKL *in vitro* experiments.

**Results:**

We found that arthritis lacking bone erosions is common in SLE patients and lupus‐prone mice. Intraarticular injection of lupus serum or IgG induces immune complex deposition and arthritis, but does not result in bone destruction. Deposition of IgG, monocytes/macrophages and TNF‐α is all required for the development of arthritis. Lupus serum or IgG inhibits RANKL‐induced differentiation of monocytes into osteoclast in a dose‐dependent manner. FcγR acts as co‐receptors for RANKL and is involved in osteoclastogenesis. Deficiency of FcγRII or FcγRIII does not affect osteoclastogenesis in the presence of SLE IgG. However, lupus IgG competes for FcγRI binding with RANKL, thereby reducing osteoclastogenesis.

**Conclusion:**

Observations from this study demonstrate that IgG from SLE patients can induce arthritis and inhibits RANKL‐induced osteoclastogenesis through competitive occupation of FcγRI on monocytes/macrophages. This study improves the understanding of the pathophysiology of SLE‐associated arthritis and offers a protective mechanism (FcγRI inhibition) that may be targeted in other forms of autoimmune/inflammatory arthritis, such as RA, to prevent or limit bone erosion and inflammatory bone loss.

## Graphical abstract

In this study, we found that lupus IgG deposition in joints can induce synovial inflammation. Recruitment of lupus IgG to FcγRI may result in functional deficiency of FcγRI on the cellular membrane, which is required for RANKL‐induced osteoclastogenesis. Thus, joint deposited lupus IgG induces arthritis while inhibiting bone erosion.

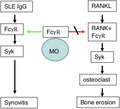

## Introduction

Systemic lupus erythematosus (SLE) is a chronic autoimmune/inflammatory disease that is characterised by the presence of autoantibodies, immune complex deposition, the activation of effector lymphocyte populations and organ damage that can affect any organ system. Depending on ethnic backgrounds, SLE affects between 1 and 45.3 per 100 000 individuals, and 80–90% of SLE patients are women of childbearing age.^1^, ^2^ Joints are involved in up to 90% of SLE patients which affects significantly patients’ quality of life.^3^, ^4^, ^5^, ^6^, ^7^

Bone erosions and inflammatory bone loss are common features in autoimmune/inflammatory arthritis, such as in rheumatoid arthritis (RA). However, they are usually absent in SLE patients and are observed in less than 5% of cases.^8^, ^9^, ^10^, ^11^ This is particularly striking, because synovial biopsies from patients with SLE demonstrate changes that are similar to those in RA, including synovial inflammation with lining cell hyperplasia.^12^, ^13^ However, inflammatory changes are less severe, and the synovial fluid contains lower number of inflammatory cells in SLE than in RA.^14^ Although tumor necrosis factor‐alpha (TNF‐α) and interleukin (IL‐)6 play a key role in RA, only serum IL‐6 levels correlate with arthritis in patients with SLE.^14^ Furthermore, high levels of autoantibodies in the serum and immunoglobulin G (IgG) and immune complex deposition in tissues are characteristic for SLE and less typical for RA.^1^, ^2^ Indeed, circulating and tissue immune complexes are centrally important for the expression of the pro‐inflammatory phenotype of SLE.^1^, ^2^ Indeed, kidney and brain damage have been linked directly to the deposition of anti‐dsDNA autoantibodies.^15^ We previously reported that monocytes/macrophages play an important role in the development of skin and liver inflammation that is induced by IgG deposition.^16^, ^17^ Furthermore, we demonstrated that monocytes/macrophage are required for inflammatory arthritis induced by bacterial DNA.^18^ However, it remains currently unknown whether and how autoantibody IgG and inflammatory cells contribute to the development of arthritis in SLE, and why bone erosions fail to develop in SLE‐associated arthritis.

Here, we investigated whether IgG deposition in the joint accounts for arthritis development and whether lupus IgG contributes to the absence of erosions. We found that intraarticular injection of lupus IgG induces arthritis but does not result in bone destruction. This is the case because lupus IgG inhibits RANKL‐induced monocyte differentiation into osteoclasts that contribute to bone erosion and inflammatory bone loss in other forms of autoimmune/inflammatory arthritis, such as RA.

## Results

### Arthritis in lupus‐prone mice is characterised by immune complex deposition and immune cell infiltration

To get a better understanding of the frequency of erosive arthritis in SLE, we analysed medical records of 1083 Chinese patients with SLE from Wuhan Union Hospital. Remarkably, we did not find evidence of joint erosions in SLE patients as assessed by plain radiographs. This indicates that lacking bone erosion is the feature of lupus arthritis.

**Figure 1:**
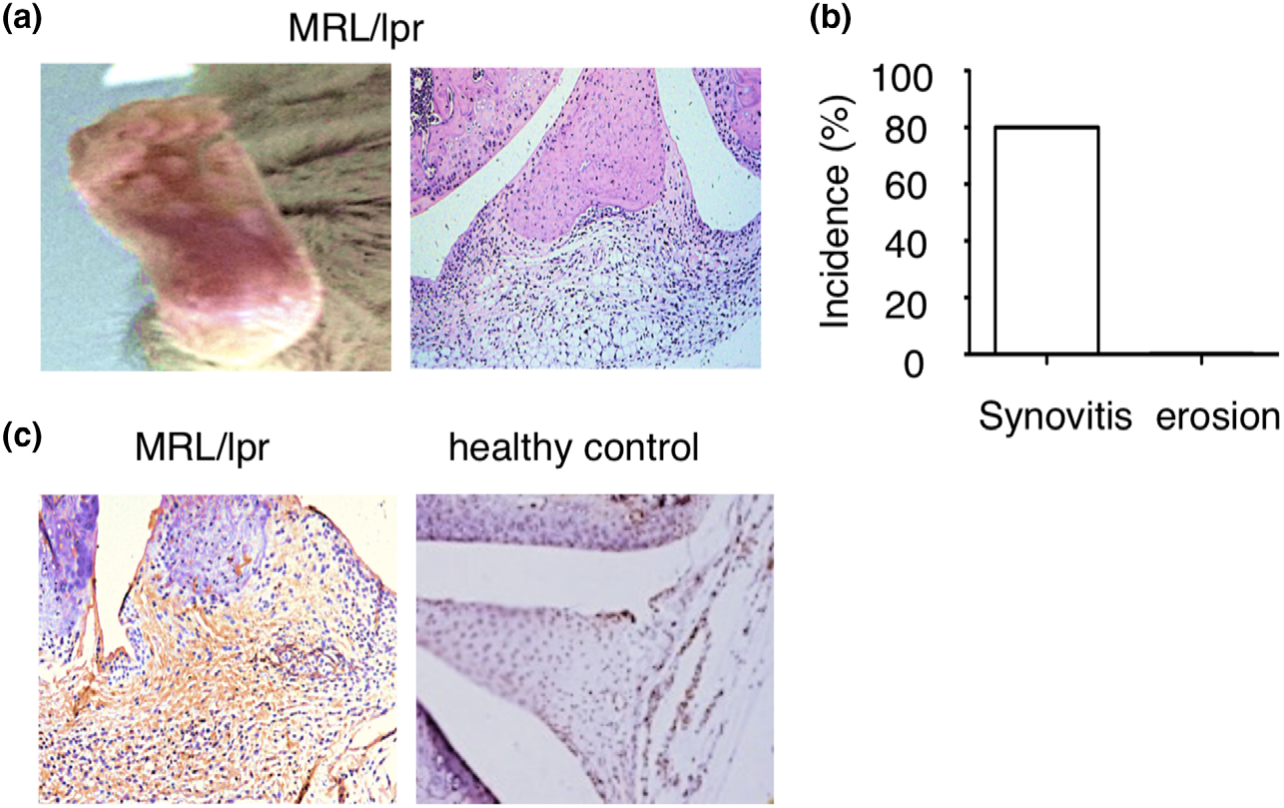
IgG deposition in arthritic joints of lupus mouse. **(a)** Representative images of ankle and histopathology of the knee joint in MRL/lpr mice at the age of 30 weeks. **(b)** Incidence of synovitis and bone erosion in MRL/lpr mice at the age of 30 weeks (*n* = 8). **(c)** Representative images of immunohistochemistry staining of IgG in the joints of MRL/lpr mice and C57BL/6 mice at the age of 25 weeks.

To further confirm that lupus arthritis lacks bone erosion, we investigated arthritic joints from lupus‐prone MRL*/lpr* mice that develop lupus‐like clinical manifestations spontaneously.^19^ We observed swollen joints in MRL/*lpr* mice at age of 30 weeks. Histopathology demonstrated inflammatory cell infiltration in the synovial tissue, but the absence of bone erosions in MRL/*lpr* mice (Figure  1a, b). These data demonstrate that lupus arthritis is characterised by synovitis lacking bone erosion.

Since tissue deposition of IgG and immune complex formation centrally contributes to tissue and organ inflammation in SLE, we investigated whether IgG is also deposited in the joints of SLE with associated arthritis. Thus, we performed immunohistochemistry to detect IgG deposition in joint from lupus MRL/lpr mice. Immunohistochemistry demonstrated IgG deposition in the joints of lupus MRL*/lpr* mice, but not in wild‐type healthy C57BL/6 mice (Figure 1c). These results indicate that IgG deposited in joints may play a critical role in the development of arthritis in SLE.

### Lupus IgG depositions cause arthritis

**Figure 2:**
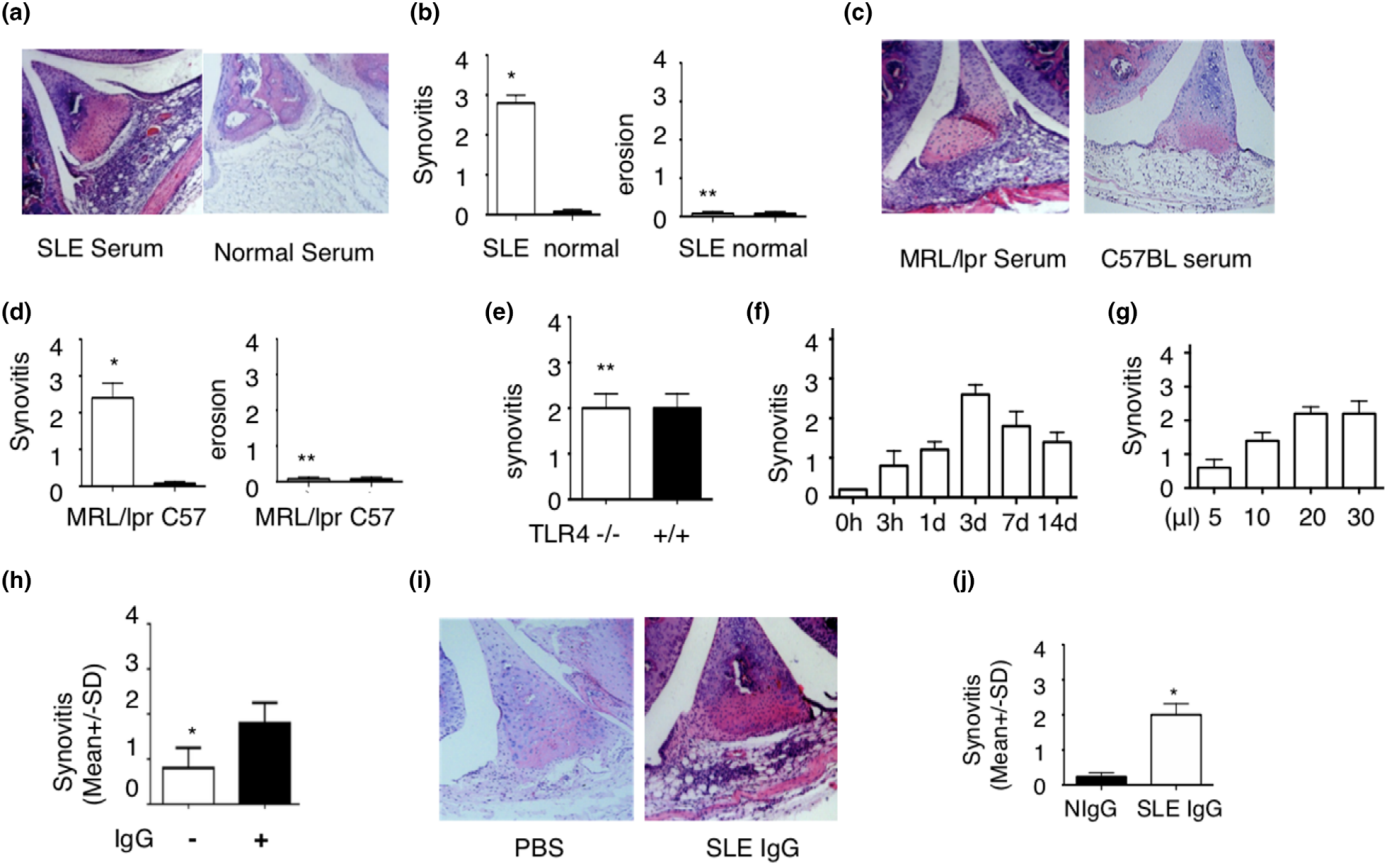
Intraarticular injection of lupus serum IgG induces arthritis. (**a**–**d**) Representative images of histopathology of knee joints **(a, c)** and severity of synovitis and bone erosion **(b, d)** in C57BL/6 mice sacrificed 3 d after intraarticular injection of 20 μL serum from lupus patients, healthy individuals, MRL/lpr mice or C57BL/6 mice. *n* = 5 per group. These experiments have been repeated at least three times. **(e)** Severity of arthritis induced by intraarticular injection of lupus serum (20 μL) in TLR4^−/−^ and TLR4^+/+^ mice. *n* = 5 per group. **(f)** Kinetics of arthritis induced by intraarticular injection of lupus serum (20 μL) in C57BL/6 mice. *n* = 5 per group. **(g)** Severity of arthritis induced by intraarticular injection of various doses of lupus serum. *n* = 5 per group. **(h)** Severity of arthritis in C57BL/6 mice sacrificed 3 d after intraarticular injection of lupus serum with or without IgG depletion (20 μL). *n* = 5 per group. **(i)** Histopathology of knee joints in C57BL/6 mice sacrificed 3 d after intraarticular injection of IgG isolated from serum of SLE patients with arthritis (50 μg in 20 μL PBS) and PBS (20 μL). **(j)** Severity of arthritis in C57BL/6 mice sacrificed 3 d after intraarticular injection of lupus serum IgG (50 μg) and normal serum IgG (50 μg). *n* = 5 per group. These experiments have been repeated at least three times. *P < 0.05, **P > 0.05.

Because of aforementioned IgG deposition in arthritic joints of SLE patients and lupus‐prone MRL/*lpr* mice, we speculated that IgG deposition may be a central contributor to the development of lupus arthritis. Thus, we induced IgG deposition in joints by intraarticular injection of serum from SLE patients into knee joint of normal C57BL/6 mice. Serum from SLE patients induced arthritis in mice without causing bone erosions, whereas serum from healthy controls did not cause inflammation (Figure 2a, b). To further investigate the role of SLE serum in the induction of arthritis, we collected serum from lupus‐prone MRL/lpr mice and wild‐type controls. We found that serum from MRL/*lpr* mice induced arthritis without causing bone erosions, but serum from wild‐type C57BL/6 mice did not (Figure 2c, d). To further determine that arthritis was caused by lupus serum but not LPS contamination, we used LPS‐resistant TLR4^−/−^ mice and LPS‐responder control mice (TLR4^+/+^). We found that a similar severity of arthritis is developed in LPS‐resistant TLR4^−/−^ mice and LPS‐responder control mice (Figure 2e). This result indicates that arthritis is induced by lupus serum not LPS contamination.

To determine when arthritis develops and how long it lasts for after intraarticular injection of lupus serum, we investigated kinetics of arthritis induced by lupus serum. We observed that arthritis occurred 3 h after injection, peaked after 3 days and lasted for at least 14 days (Figure 2f). To evaluate whether severity of arthritis is related to the dose of lupus serum injected, we injected variable amounts of lupus serum. We found that the severity of the arthritis was dose‐dependent (Figure 2g).

Since a high level of IgG exists in lupus serum, but is not the only potential pathogenic factor, we chose to deplete SLE serum from IgG prior to intraarticular injection. We used protein G agarose to deplete IgG from lupus serum and found that synovial inflammation was significantly reduced in mice in response to intraarticular injection of IgG‐depleted lupus serum when compared to ‘full’ lupus serum without IgG depletion (Figure 2h). To confirm whether lupus IgG directly induces arthritis, we injected intraarticularly IgG isolated from lupus serum into wild‐type mice and found that lupus IgG directly induced immune complex deposition and arthritis (Figure 2i). These data indicate that IgG deposited in the joint can instigate the development of lupus arthritis. We also compared severity of arthritis induced by the same dose of IgG from lupus serum and healthy serum and found that IgG from lupus serum induced more severe synovitis than IgG from healthy serum (Figure 2j).

### Lupus IgG depositions cause arthritis through monocytes/macrophages and TNF

**Figure 3:**
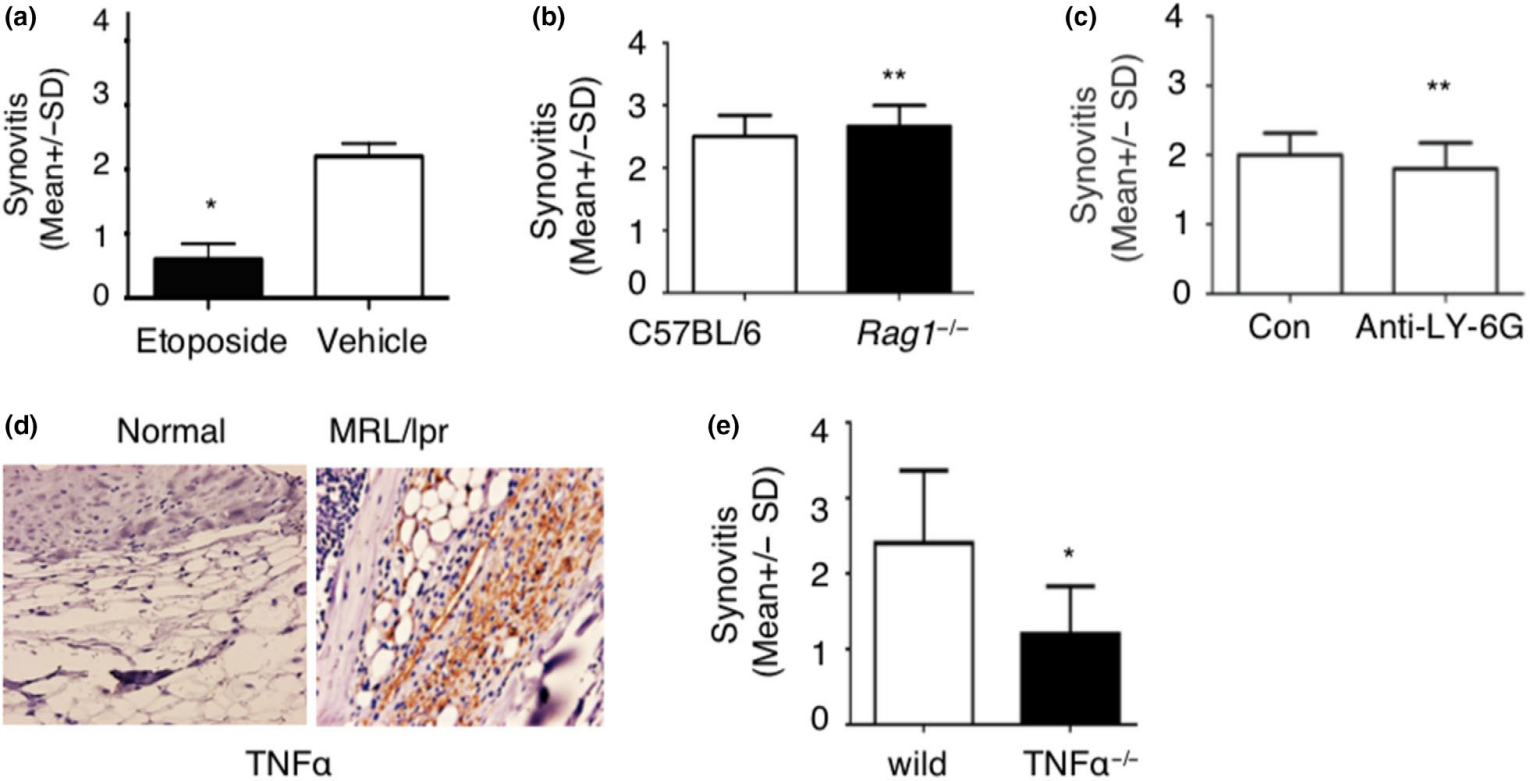
Lupus serum IgG causes arthritis through monocytes/macrophages and TNF. **(a)** Severity of arthritis induced by intraarticular injection of lupus serum IgG in C57BL/6 mice with or without treatment of etoposide. *n* = 5 per group. **(b)** Severity of arthritis in *Rag1*‐deficient mice and C57BL/6 mice with intraarticular injection of lupus serum IgG. *n* = 5 per group. **(c)** Severity of arthritis induced by intraarticular injection of lupus serum IgG in C57BL/6 mice with or without depletion of neutrophils (anti‐Ly‐6G). *n* = 5 per group. **(d)** Immunohistochemistry staining of TNF‐α in the joints of MRL/lpr mice and C57BL/6 mice at the age of 25 weeks. **(e)** Severity of arthritis induced by intraarticular injection of lupus serum IgG in TNF‐α‐deficient mice and wild‐type mice. *n* = 5 per group. These experiments have been repeated at least two times. Data are represented as mean ± SD. Dose of intraarticular injection of lupus IgG is 50 μg. *P < 0.05, **P > 0.05.

Monocytes/macrophages are required for the development of skin and liver inflammation induced by lupus IgG.^16^, ^17^ Thus, we aimed to determine the role of monocytes/macrophages in the development of arthritis induced by lupus IgG using etoposides which selectively deplete monocytes/macrophages. Monocyte depletion resulted in significantly reduced arthritis in mice (Figure 3a). In addition, we also determined the role of lymphocytes in the development of arthritis induced by lupus IgG through using *Rag 1*‐deficient mice that lack mature T and B cells but have intact monocytes/macrophages.^16^ We found that development of arthritis was not affected in *Rag 1*‐deficient mice (Figure 3b). Interestingly, the depletion of neutrophils with anti‐Ly‐6G antibody^18^ did not affect the development of this arthritis (Figure 3c). These data indicate that monocytes/macrophages play an important role in the development of arthritis induced by joint deposited lupus IgG.

Since TNF is an important pro‐inflammatory cytokine and plays a key role in pathogenesis of inflammatory arthritis,^18^, ^20^ we investigated the role of TNF in arthritis induced by lupus serum IgG. Immunohistochemistry staining demonstrates that a large amount of TNF is expressed in arthritic joints in lupus MRL/lpr mice (Figure 3d). Furthermore, we used TNF‐α‐deficient mice and found that severity of arthritis was significantly decreased in TNF‐α‐deficient mice compared to wild‐type mice (Figure 3e). These results support that TNF‐α exerts a key role in the development of arthritis induced by lupus serum IgG.

### Lupus serum IgG inhibits RANKL‐induced osteoclastogenesis

We previously reported that lupus IgG promotes monocyte differentiation into dendritic cells.^16^ However, monocytes can also differentiate into osteoclasts in the presence of RANKL.^20^ Thus, we speculated that lupus IgG may inhibit RANKL‐induced monocyte differentiation into osteoclasts, for example, through promotion of differentiation into dendritic cells. This may be the case since considerable amount of lupus IgG deposits in the synovial tissue of SLE patients.

**Figure 4:**
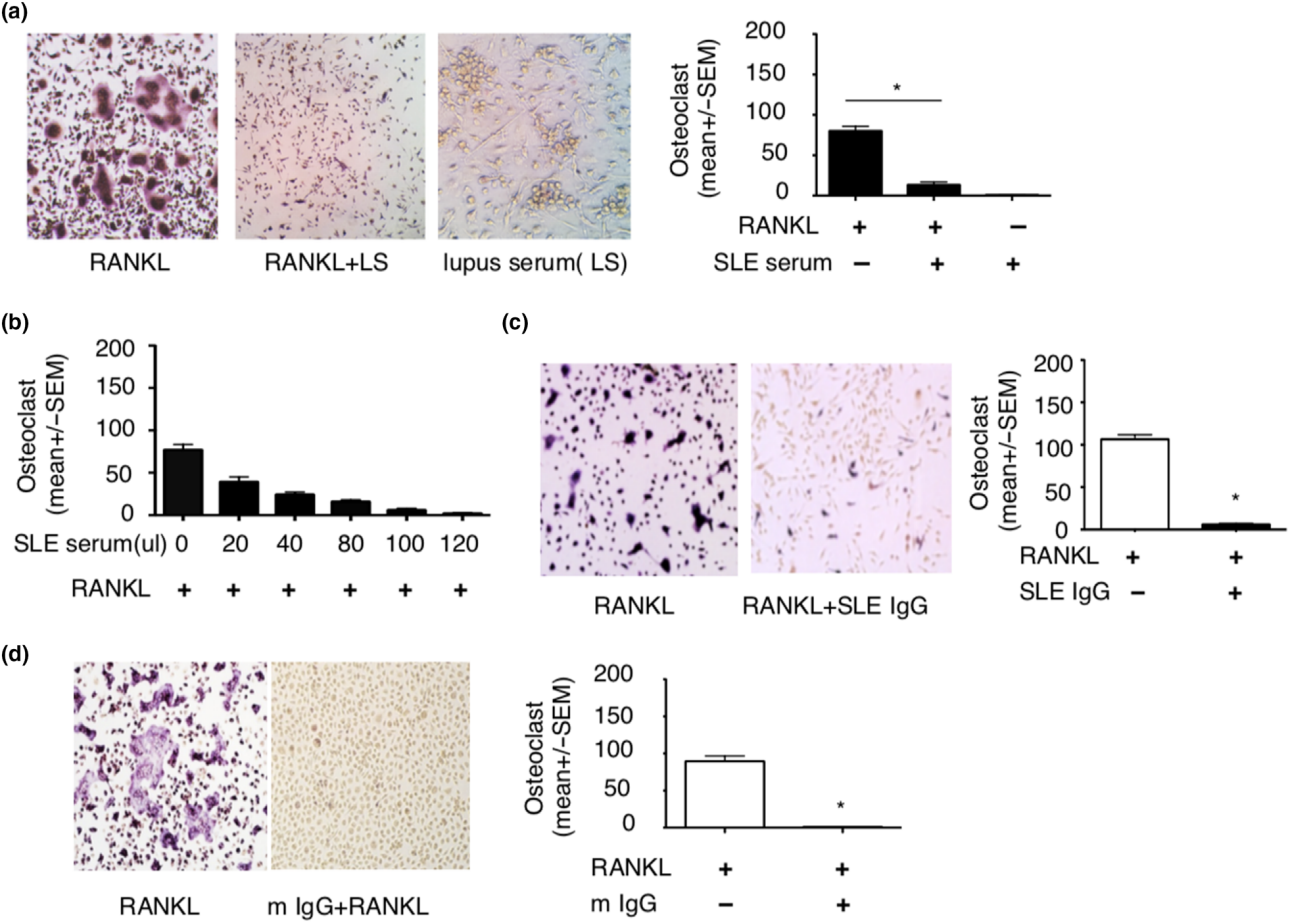
Lupus serum IgG inhibits RANKL‐induced osteoclastogenesis. **(a)** Photographs and number of TRAP‐positive osteoclasts in bone marrow macrophages (BMMs) treated with RANKL (100 ng), RANKL (100 ng) + lupus serum (LS, 80 µL) and lupus serum (LS, 80 µL), respectively. **(b)** Number of TRAP‐positive osteoclasts in BMMs treated with RANKL (100 ng) in the presence of various doses of lupus serum. **(c)** Photographs and number of TRAP‐positive osteoclasts in BMMs incubated with RANKL (100 ng) in the absence or presence of SLE IgG (100 µg). **(d)** Photographs and number of TRAP‐positive osteoclasts in BMMs treated with RANKL (100 ng) in the presence of SLE IgG (100 µg) isolated from lupus MRL/lpr mice serum (m IgG). These experiments have been repeated at least three times. Data are represented as mean ± SEM. Group with RANKL compared with group with RANKL + SLE serum or IgG, *P < 0.05.

To test our hypothesis, we treated bone marrow monocytes/macrophages (BMMs) with RANKL to generate osteoclasts in the presence or absence of lupus serum and evaluated the development of TRAP‐positive cells which represent osteoclast.^20^ Lupus serum significantly inhibited RANKL‐induced osteoclastogenesis, but lupus serum alone induced the development of TRAP‐negative cells (Figure 4a). To determine whether inhibitory effects of lupus serum on osteoclastogenesis are related to dose of lupus serum, we used various doses of lupus serum. We found that suppression of osteoclastogenesis depended on the dose of lupus serum (Figure 4b).

Next, we determined whether lupus IgG is the major component to inhibit osteoclastogenesis using IgG isolated from serum of lupus patients. Indeed, we found that lupus IgG directly suppressed RANKL‐induced osteoclastogenesis (Figure 4c). To further confirm that IgG from lupus mice can inhibit osteoclastogenesis, we also used IgG from lupus MRL/*lpr* mice and found that IgG from lupus MRL/*lpr* mice also inhibited RANKL‐induced osteoclastogenesis (Figure 4d). Taken together, these data indicate that lupus IgG can inhibit RANKL‐induced osteoclastogenesis.

### FcγRII and FcγRIII are not involved in inhibitory effects of lupus IgG on RANKL‐induced osteoclastogenesis

The Fcγ receptor (FcγR) is not only a receptor for IgG, but is also a costimulatory molecule for RANKL‐induced osteoclastogenesis,^21^, ^22^ and FcγR includes FcγRI, FcγRII and FcγRIII. Thus, we determined the role of FcγRII and FcγRIII in inhibitory effect of lupus IgG on osteoclastogenesis using FcγRII‐ and FcγRIII‐deficient mice.

**Figure 5:**
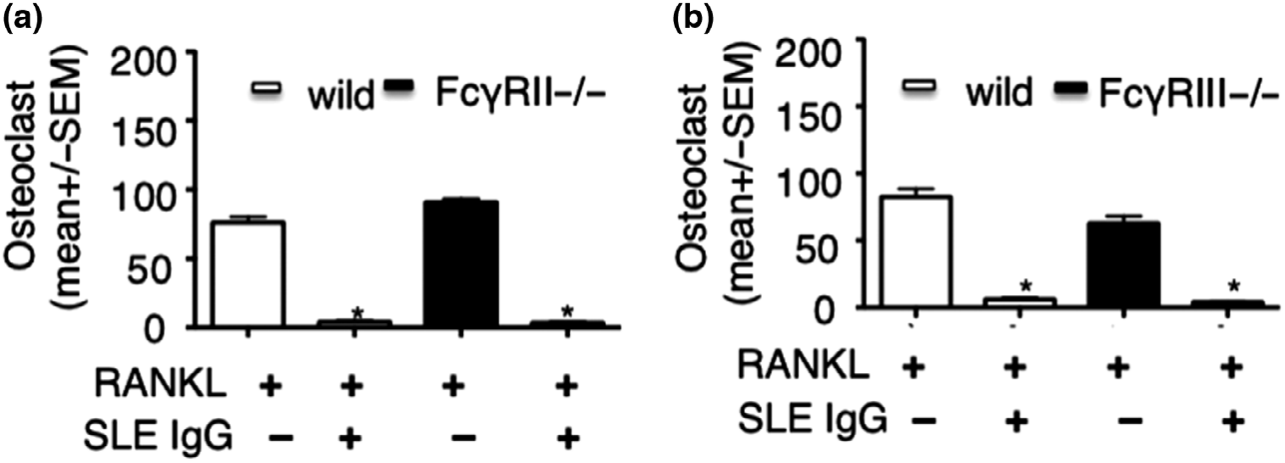
The role of FcγRII and FcγRIII in the inhibitory effect of lupus IgG on RANKL‐induced osteoclastogenesis. **(a)** Number of TRAP‐positive osteoclasts in BMMs from wild‐type and FcγRIIB^−/−^ mice incubated with RANKL (100 ng) in the absence and the presence of SLE IgG (100 μg). **(b)** Number of TRAP‐positive osteoclasts in BMMs from wild‐type and FcγRIII^−/−^ mice incubated with RANKL (100 ng) in the absence and the presence of SLE IgG (100 μg). These experiments have been repeated at least three times. Data are represented as mean ± SEM. Group with RANKL compared with group with RANKL + SLE IgG, *P < 0.05.

First, we isolated bone marrow monocytes (BMMs) from FcγRII‐deficient mice to determine the role of FcγRII during the inhibition of osteoclastogenesis through lupus IgG. RANKL induced more osteoclastogenesis in FcγRIIB‐deficient monocytes when compared to wild‐type monocytes (Figure 5a). However, RANKL‐induced osteoclastogenesis was inhibited by lupus IgG in FcγRIIB‐deficient monocytes similar to wild‐type monocytes (Figure 5a). This indicates that FcγRIIB does not play a central role in the inhibitory effect of lupus IgG on RANKL‐induced osteoclastogenesis.

Next, we used FcγRIII‐deficient mice to determine the role of FcγRIII in the inhibition of osteoclastogenesis through lupus IgG. In RANKL‐induced osteoclastogenesis, the number of osteoclast was slightly reduced using cells from FcγRIII‐deficient when compared to wild‐type mice (Figure 5b). This indicates that FcγRIII may exert a key role in RANKL‐induced osteoclastogenesis. Furthermore, we found that lupus IgG inhibits osteoclastogenesis from FcγRIII‐deficient monocytes induced by RANKL. Results were comparable using monocytes from FcγRIII‐deficient or wild‐type animals (Figure 5b). This suggests that FcγRIII is involved in osteoclastogenesis but does not mediate inhibitory effects of lupus IgG on RANKL‐induced osteoclastogenesis. Thus, these results suggest that FcγRI may be key in this process.

### Lupus IgG reduces FcγRI, but not FcγRII and FcγRIII surface expression on monocytes

**Figure 6:**
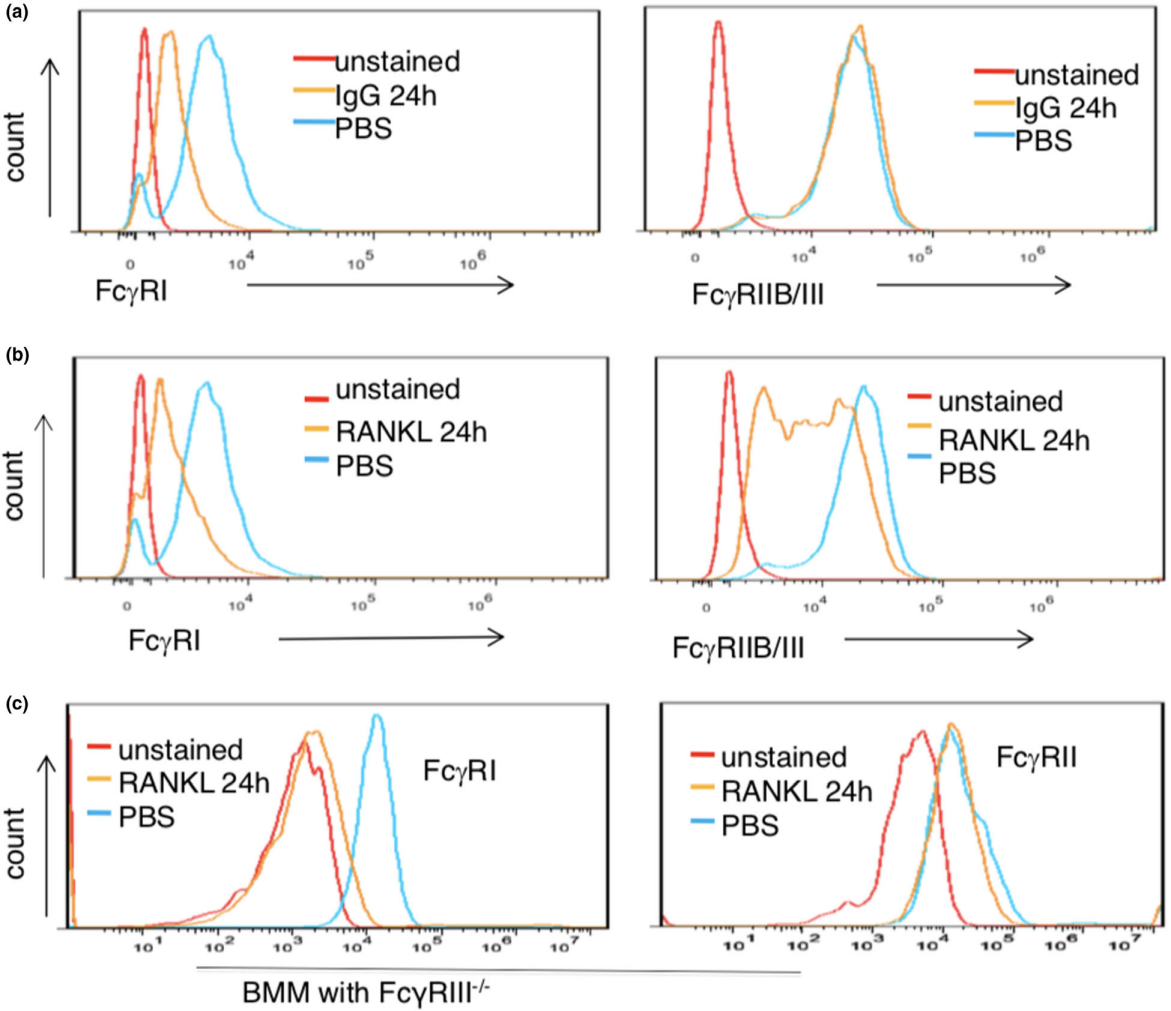
Lupus IgG and RANKL reduce the level of FcγRI on BMMs. **(a)** Flow cytometry detected levels of CD64 (FcγRI) and CD16/CD32 (FcγRIIB/III) on BMMs treated with lupus IgG (100 μg) and PBS for 24 h. **(b)** Flow cytometry detected expression of CD64 (FcγRI) and CD16/CD32 (FcγRIIB/III) on BMMs treated with RANKL (100 ng) and PBS for 24 h. **(c)** Flow cytometry detected expression of CD64 (FcγRI) and CD16/CD32 (FcγRIIB/III) on FcγRIII‐deficient BMMs treated with RANKL (100 ng) and PBS for 24 h. Unstained means that no staining antibody was added. These experiments have been repeated at least three times.

To understand how the inhibitory effect of lupus IgG on osteoclastogenesis relates to FcγRs, we used flow cytometry to quantify surface expression of FcγRI and FcγRII and FcγRIII on monocytes in the absence or presence of lupus IgG. Surface expression of FcγRI on monocytes significantly decreased after lupus IgG treatment when compared with control PBS. Of note, lupus IgG did not affect the level of FcγRII/III surface expression on monocytes (Figure 6a). Data indicate that lupus IgG can decrease FcγRI expression on monocytes.

To determine whether RANKL regulates the level of FcγR on monocytes, we also measured surface expression of FcγRI and FcγRIIB/III on monocytes in the presence or absence of RANKL. Indeed, RANKL significantly downregulated FcγRI and FcγRIIB/III expression on monocytes as compared to control PBS (Figure 6b).

Since only antibody recognised both FcγRIIB and FcγRIII but no antibody for FcγRIIB or FcγRIII is available, reduced levels of FcγRIIB/III on monocytes may be caused by decreased FcγRIIB or FcγRIII. Thus, we used FcγRIII‐deficient monocytes to further investigate effects of RANKL on expression of FcγRI and FcγRII. RANKL significantly reduced the level of FcγRI expression on FcγRIII‐deficient monocytes, but RANKL did not affect level of FcγRII on FcγRIII‐deficient cells (Figure 6c).

The data demonstrate that RANKL significantly reduces FcγRI expression on monocytes. Furthermore, it slightly reduces the level of FcγRIII surface expression.

### SLE IgG inhibits RANKL‐induced osteoclastogenesis through occupation of FcγRI

Since both IgG and RANKL can reduce the level of FcγRI surface expression on monocytes, and since FcγRI is required for both IgG and RANKL signal transduction,^21^, ^22^ we speculated that IgG and RANKL may suppress each other through competition for FcγRI binding.

**Figure 7:**
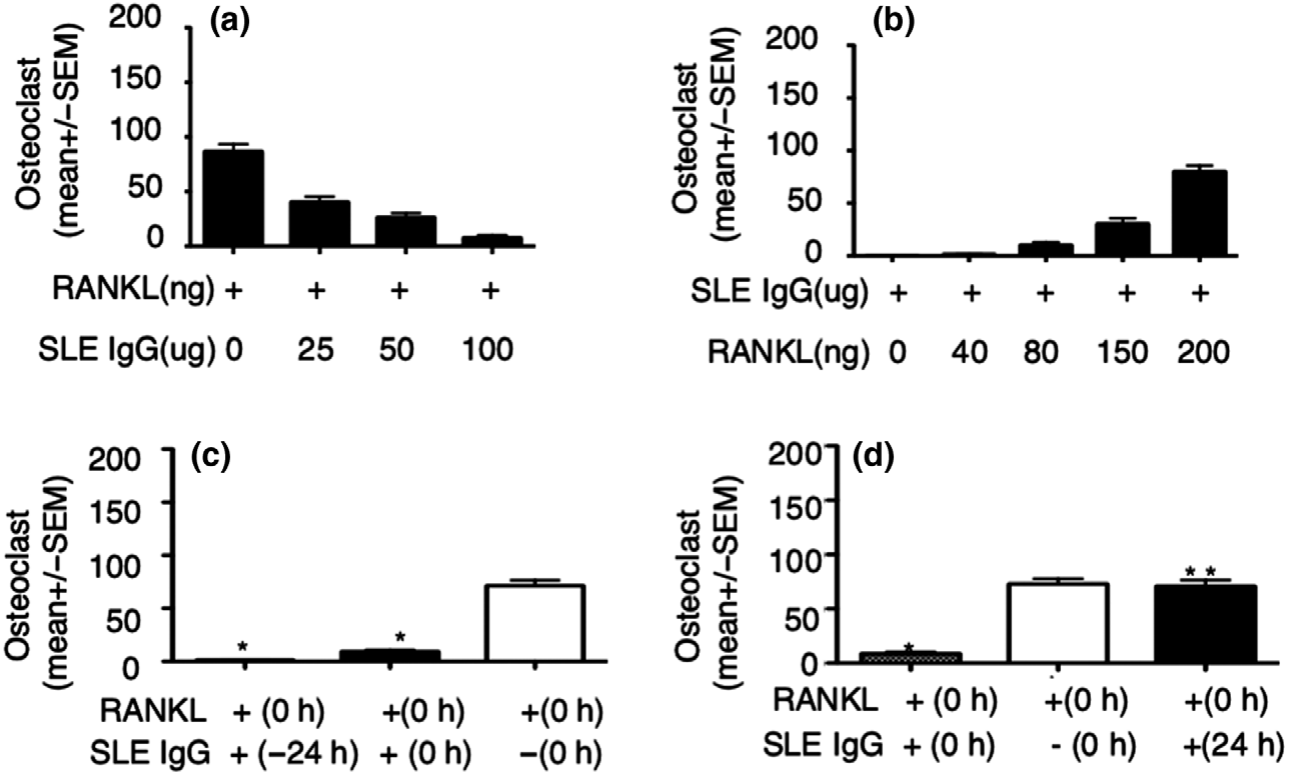
Lupus IgG inhibits RANKL‐induced osteoclastogenesis through occupied FcγRI. **(a)** Number of TRAP‐positive osteoclasts in BMMs treated with RANKL (100 ng) in the presence of various doses of SLE IgG. **(b)** Number of TRAP‐positive osteoclasts in BMMs treated with lupus IgG (100 µg) in the presence of various doses of RANKL. **(c, d)** Number of TRAP‐positive osteoclasts in BMMs treated with SLE IgG (100 µg) at various time before or after RANKL (100 ng) stimulation. +(−24 h) means that SLE IgG was added 24 h before RANKL, +(24 h) means that SLE IgG was added 24 h after RANKL stimulation, +(0 h) means that SLE IgG and RANKL were added at the same time, and −(0 h) means no SLE IgG treatment. These experiments have been repeated at least three times. Data are represented as mean ± SEM. Group with RANKL compared with group with RANKL + SLE IgG, *P < 0.05; **P > 0.05.

Thus, we investigated whether the inhibitory effect of lupus IgG is related to its dose. To this aim, we used the certain dose of RANKL and various doses of lupus IgG in the experiment. Monocytes were treated with RANKL and various doses of lupus IgG at the same timepoint. High doses of lupus IgG displayed a stronger inhibitory effect on osteoclastogenesis as compared to lower doses of lupus IgG (Figure 7a).

Next, we investigated whether the inhibitory effect of lupus IgG on osteoclastogenesis can be blocked by the high doses of RANKL. We used a constant dose of lupus IgG and various doses of RANKL. Inhibitory effects of lupus IgG on osteoclastogenesis were gradually blocked by increasing doses of RANKL (Figure 7b) suggesting competition between molecules.

Third, we determined whether inhibitory effects of lupus IgG on osteoclastogenesis depend on the extent of IgG binding to FcγRI. Thus, we treat monocytes with lupus IgG 24 h before stimulation with RANKL. We observed stronger inhibitory effects of lupus IgG on RANKL‐induced osteoclastogenesis in cells pretreated for 24 h than in cells stimulated with both RANKL and IgG at the same time (Figure 7c).

Lastly, we evaluated whether the inhibitory effect of lupus IgG on osteoclastogenesis is blocked by the extent of RANKL binding to FcγRI. Thus, we used lupus IgG to treat monocytes 24 h later than RANKL stimulation. We found that inhibitory effect of lupus IgG on osteoclastogenesis was converted at 24 h after RANKL stimulation (Figure 7d).

Taken together, these data suggest that lupus IgG inhibits RANKL‐induced osteoclastogenesis through competition for FcγRI binding.

## Discussion

This study and others^5^, ^6^, ^7^, ^8^, ^9^, ^10^, ^11^, ^12^ demonstrate that arthritis is common in SLE but does not cause significant erosion and/or inflammatory bone loss.

Here, we show that IgG deposition in the joint plays a crucial role in the development of lupus‐associated arthritis in humans and mice. This is in line with the observation that IgG deposition in tissues and organs is an important pathophysiological feature in SLE,^1^ contributing to damage in the kidney, skin and other organs.^15^, ^20^ Arthritis was established in wild‐type mice through intraarticular injection of lupus serum or lupus IgG. Previously, it has been demonstrated that IgG receptor‐deficient mice, including FcγR‐deficient animals, fail to develop collagen‐induced arthritis^23^ offering further arguments supporting IgG deposition causing arthritis.

In most cases, autoimmune/inflammatory arthritis causes bone erosion and destruction. However, lupus‐associated arthritis is usually painful, can be severe, but is usually not destructive. Although previous studies demonstrate the effects of immune complexes on RANKL‐induced osteoclastogenesis,^24^, ^25^, ^26^, ^27^, ^28^ the role of lupus IgG on bone destruction in lupus arthritis is not clear.

The present study demonstrates that lupus IgG inhibits RANKL‐induced osteoclastogenesis, suggesting that joint deposited lupus IgG can prevent bone destruction in lupus arthritis. Our data display IgG deposition in joints of lupus patients and mice. Intraarticular injection of lupus IgG induced arthritis in the absence of bone erosions and/or inflammatory bone loss. Lupus IgG directly inhibited RANKL‐induced osteoclastogenesis in a dose‐dependent manner *in vitro*. Inhibitory effects of lupus serum on osteoclastogenesis are not caused by other factors, such as remainders of anti‐inflammatory medication in the serum, because serum and also purified IgG from the same SLE patient induced arthritis *in vivo* while inhibiting osteoclastogenesis *in vitro*. This strongly suggests that lupus IgG deposited in the joint is an important contributor to inflammation while preventing bone destruction.

Several lines of evidence strongly support that lupus IgG inhibits RANKL‐induced osteoclastogenesis through competing for binding to FcγRI. Of note, FcγRI is required for IgG‐ and RANKL‐mediated signal transduction, and both lupus IgG and RANKL can significantly reduce FcγRI surface expression on monocytes *in vitro*. We excluded the possibility that lupus IgG affects flow cytometry antibody binding to FcγRI, because binding of this antibody to mouse FcγRI is not inhibited by human IgG.^29^ Since both lupus IgG‐ and RANKL‐mediated signal transduction require FcγRI, the observation that lupus IgG and RANKL compete for FcγRI is a very central observation. Lupus IgG binding to FcγRI may result in functional deficiency of FcγRI that is required for RANKL‐induced osteoclastogenesis. In contrast, RANKL‐induced osteoclastogenesis may also lead to functional deficiency of FcγRI that is required for IgG signalling transduction. Data presented here show that lupus IgG has stronger inhibitory effect on osteoclastogenesis at 24 h prior to RANKL stimulation, and lupus IgG loses inhibitory effect on osteoclastogenesis at 24 h after RANKL stimulation. Thus, the presence of IgG in SLE patients may have long‐standing inhibitory effects on RANKL recruitment to FcγRI and osteoclastogenesis. Conversely, deficiency of FcγRIII and FcγRIIB did not significantly block inhibitory effect of lupus IgG on osteoclastogenesis, suggesting that effects are specific to FcγRI. This is in line with the observation that activating FcγR, but not inhibitory FcγR, is decreased on osteoclasts as compared to monocytes/macrophages.^26^

We found that TNF plays important role in the development of arthritis induced by lupus IgG, but this result does not suggest that TNF is the therapeutic target in SLE patients. TNF has two functions including induction of NF‐kB activation and induction of apoptosis. It has been shown that recombinant TNF‐alpha induces a significant delay in the development of the nephritis in lupus mice.^30^ TNF may exert the role of inhibiting development of SLE at the early stage because of inducing apoptosis and plays the role of promoting the progress of SLE at the late stage because of activating NF‐kB in lupus mice. Since there is the problem of cell apoptosis in SLE patients, TNF inhibitors and Fc receptor blockers in treatment of RA patients may induce production of ANA and dsDNA Ab.^31^ However, kidney injury is closely related to deposition of dsDNA in kidney tissue in SLE patients; thus, TNF inhibitors and Fc receptor blockers are not used for treatment of SLE patients. Furthermore, TNFR1 blocker promoted kidney damage in lupus‐prone mice.^32^

Although offering a new pathomechanism of SLE‐associated arthritis, this study has limitations. For instance, we did not investigate the molecular mechanism by which lupus IgG reduces the level of FcγRI surface expression on monocytes/macrophages and lupus IgG results in functional deficiency of FcγRI required for RANKL‐induced osteoclastogenesis.

### Conclusions

**Figure 8:**
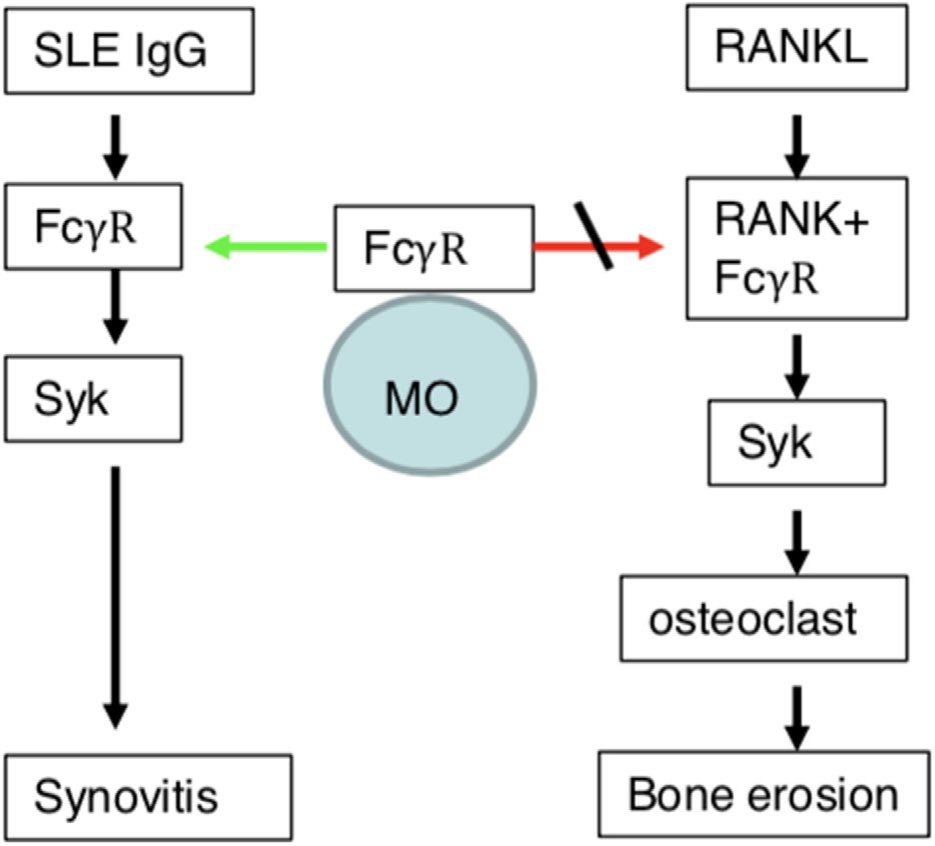
Lupus IgG inhibits RANKL‐induced osteoclastogenesis. Lupus IgG deposition in the joints can mediate synovial inflammation. Recruitment of lupus IgG to FcϒRI may result in functional deficiency of FcϒRI on the cellular membrane, which is required for RANKL‐induced osteoclastogenesis.

IgG deposition in joints can induce synovial inflammation. Recruitment of lupus IgG to FcγRI may result in functional deficiency of FcγRI on the cellular membrane, which is required for RANKL‐induced osteoclastogenesis. Thus, IgG induces inflammation while inhibiting erosion and inflammatory bone loss (Figure 8). This study transforms the understanding of the pathomechanism of non‐destructive arthritis in SLE. It promises potential for new therapeutic approaches to other forms of autoimmune/inflammatory arthritis (such as RA) through FcγRI inhibition.

## Methods

### SLE sera

Lupus serum and normal sera were provided by Department of Rheumatology, Wuhan Union Hospital, affiliated to Tongji Medical College and the First Affiliated Hospital of Nanjing Medical University. Sera were collected from B6.MRL/lpr, MRL/lpr and normal C57BL/6 mice.

Medical records of patients with SLE from Department of Rheumatology, Union Hospital, affiliated to Tongji Medical College were retrospectively analysed between 1 January 2016 and 2 April 2019. The study was approved by Institutional Review Board of Tongji Medical College (2019IECS1015) and Nanjing Medical University (IACUC‐14030140).

### Mice and reagents

C57BL/6 mice were purchased from the Animal Center of Nanjing Medical University. B6.MRL/*lpr* and TLR4^−/−^ mice were purchased from the Model Animal Research Center of Nanjing University (Nanjing China). FcγRIIB^−/−^, FcγRIII^−/−^ and MRL/*lpr* mice were purchased from Jackson Laboratories (Bar Harbor, ME, USA). Mice of 6 to 8 weeks old were used in all the experiments. All mice were housed within the Animal Center of Nanjing Medical University and Tongji Medical College. Protein G agarose was purchased from HyClone.

### Intraarticular injection

After shaving and disinfection of the injection area of anaesthetised mice, 20 µL of lupus serum, lupus IgG (50 µg or 100 µg in 20 µL), normal human serum or PBS was injected intraarticularly into mice using a disposable insulin syringe.^18^, ^20^

### Examination of histopathology and immunohistochemistry

The joints were collected from experimental mice. After routine fixation and paraffin embedding of joints, the tissues were cut to 5 µm thickness. Afterwards, histopathological and immunohistochemistry staining was performed on the joint tissue; the severity of the arthritis was scored from 0 to 4.^18^, ^20^

### *In vitro* osteoclastogenesis

Bone marrow monocytes (BMMs) extracted from the tibias of 4‐week‐old mice were cultured in the presence of 50 ng M‐CSF (Peprotech) for 3 days; then, 100 ng receptor activator of nuclear factor kappa B ligand (RANKL, Peprotech) was added for 3–4 days to generate osteoclasts. At the indicated time points, SLE serum and SLE IgG were added. Cultures were then fixed and stained for tartrate‐resistant acid phosphatase (TRAP) according to the manufacturer’s procedure (Sigma).^20^

### Flow cytometry detection of FcγR on BMMs

Bone marrow monocytes (BMMs) extracted from the tibias of 4‐week‐old mice were cultured in the presence of 50 ng M‐CSF (Peprotech) for 3 days. BMMs were treated with SLE IgG or RANKL for different time, then were stained with specific antibodies for FcγRI (PE‐conjugated, BD) and FcγRIIB/FcγRIII (FITC‐conjugated, BD) and analysed with a CytoFLEX Flow Cytometer (Beckman).

### Statistical analysis

Statistical analyses were performed using a two‐tailed Student’s *t*‐test for two groups. *P* ﹤ 0.05 is considered to be statistically significant.

## Conflicts of interest

The authors declare no conflict of interest.

## Author contributions

GM Deng: Design of research plan and experiments; analysis of results; writing of the manuscript. WQ, YZ, LJ, HD: conduction of major experiments; analysis of results. JZ, LY, RD, CH: Contribution of data for the manuscript.
